# Key Steps in Murine Cardiac Engineering Validation: How Does It Affect the Efficiency of Cardiac Devices?

**DOI:** 10.1155/bmri/5247814

**Published:** 2026-03-25

**Authors:** Nancy G. Viveros-Moreno, X. Fernanda Rodriguez-Reyes, Mario Garcia-Lorenzana, Marcela Salazar-García, Laura Villavicencio-Guzmán, Nohra E. Beltran-Vargas

**Affiliations:** ^1^ Postgraduate Program in Biological and Health Sciences, Universidad Autónoma Metropolitana, Mexico City, Mexico, uam.mx; ^2^ Biological Engineering Undergraduate Program, Division of Natural Science and Engineering, Universidad Autónoma Metropolitana, Mexico City, Mexico, uam.mx; ^3^ Department of Reproduction Biology, Division of Biological and Health Sciences, Universidad Autónoma Metropolitana, Mexico City, Mexico, uam.mx; ^4^ Research Laboratory of Developmental Biology and Experimental Teratogenesis, Hospital Infantil de Mexico, Mexico City, Mexico; ^5^ Department of Processes and Technology, Division of Natural Sciences and Engineering, Universidad Autónoma Metropolitana, Mexico City, Mexico, uam.mx

**Keywords:** acute myocardial infarction, cardiac patch, cardiac regenerative medicine, cardioprotection, preclinical validation

## Abstract

Acute myocardial infarction is one of the leading causes of death in the world. Owing to the limited regenerative capacity of the heart, the development of therapeutic devices using cell therapy and tissue engineering has gained importance. Preclinical validation of these devices can provide evidence of their safety and efficacy in implantation in infarcted myocardium. In view of the growing number of proposed devices, it is necessary to establish an appropriate experimental protocol that mimics the clinical scenario. This review is aimed at describing the strategies used for the preclinical validation of an artificial myocardium, based on the purpose of the therapy and the experimental design. Overall, the development of cardiac devices has focused on in vitro or in situ strategies for cell delivery to the infarcted tissue and proper cell proliferation and differentiation. Efforts have also been made to search for new cell sources, blood vessel formation, development of an anti‐inflammatory phenotype, and electromechanical coupling of resident and implanted cardiomyocytes. Based on the published evidence, a seven‐stage process for the preclinical validation of cardiac regenerative therapy is proposed.

## 1. Introduction

Acute myocardial infarction (AMI) is a cardiovascular disease that affects approximately 126 million people worldwide [1]. In pathologies with a poor capacity to self‐regenerate, cell therapy and tissue engineering develop artificial tissues to replace the injured area. Despite significant advances in cardiac regenerative medicine, new strategies are constantly being developed to prevent systolic dysfunction after AMI [2, 3]. These papers cover a wide range of approaches, including cell‐free, cell‐based, and combined approaches used in cardiac regenerative medicine [3]. In this review, they are grouped under the term “therapeutic device” (TD). The aim of the different TDs is to reduce the size of the infarct scar, increase myocardial viability, improve cardiac function, preserve synchronous contraction, and limit inflammatory response [4, 5].

Implanting a TD after AMI has been shown to improve the structural and functional properties of infarcted myocardium. However, the constant loss of cells during delivery, the high rate of cell death after implantation, and the failures of communication and integration between the implanted tissue and the host tissue remain a challenge. This rapid and massive loss is associated with the delivery vehicle or the mechanical force of the heartbeat. Of the remaining cells, there is a gradual loss due to cell death, hypoxia, or induction of an unfavorable immune response [6, 7]. Eventually, the cells increase in number, although they do not exceed the initial number of cells administered [4, 8]. This represents, in part, the feasibility of the proposed TD. Furthermore, it has been reported that the lack of electrical connection between the healthy myocardium and the cardiac implant may contribute to asynchronous ventricular contraction and ultimately lead to heart failure [9, 10]. Herein lies the importance of validating a TD with a methodological approach that can mimic the dynamics occurring in the infarcted myocardium.

On the other hand, the feasibility, efficacy, and safety of cell therapy [11] and tissue engineering [12] for therapeutic use must be demonstrated prior to clinical trials. Small animal studies provide initial feasibility, which should be thoroughly evaluated in large species whose cardiovascular physiology more closely resembles that of humans. The correct choice of animal model and appropriate experimental design play a key role in preclinical research [13]. Experimental designs aimed at validating TDs require the validation of an animal model that corresponds to the clinical entity, as well as the adequate systematization of the variables to be evaluated. The lack of systematization in the experimental design could affect the choice of the device with the greatest benefits for its transfer to clinical trials. It has been documented that just only 5%–10% of animal studies are translatable to human response [14, 15]. Improvements in experimental design optimize clinical translation by ensuring that preclinical research findings are reliable, rigorous, and reproducible. A good preclinical experimental design considers clinically relevant variables and outcomes, thereby reducing barriers to its application in patients.

The value of preclinical research mainly conducted in animal model experiments to predict the efficacy of therapies and treatment strategies in human trials remains contentious, as most research findings are irreproducible [14, 15]. Therefore, it is necessary to recommend a unifying methodological criterion for the preclinical validation of cardiac tissue engineering that allows the evaluation of the functionality of therapies with comparable results in order to move on to clinical trials (e.g., the identification of specific biomarkers, definition of the initial size of the infarct, or functional improvements).

This paper describes the strategies reported for the validation of a functional artificial myocardium, based on the development of the in vivo murine model, the selection of the variables to be studied after implantation, and the current status of the TDs reported in preclinical studies, in order to establish a framework for research groups interested in preclinical validation of cardiac TDs.

## 2. Methodology

### 2.1. Search Strategy

The search for original articles from 2013 to December 2023 was conducted in the PubMed, Web of Science, and Scopus databases. Controlled vocabulary (e.g., myocardial infarction) and keywords (e.g., left ventricular ejection fraction [LVEF]) were used for the search (Table 1).

**Table 1:** Search terminology.

Term	Keyword
Myocardial infarction	Heart
	Heart failure (HF)
	Ventricular regeneration
	Cardiac Regeneration
	Left ventricular ejection fraction (LVEF, FEVI)
	Fractional shortening (FS)
Tissue engineering	Scaffold
	Biomaterials
	Patch
	Hydrogel
	(scaffold OR hydrogel OR patch OR biomaterial)
	(collagen OR gelatin OR acid hyaluronic OR alginate OR agarose OR chitosan OR queratin OR matrigel OR decellularizad OR fibrina OR PGA OR PLLA OR PHA)
Cell therapy	Stem cell
	(progenitor∗ or cardi∗ or heart∗ or stem∗ cell∗ or myocyt∗)
Preclinical	Animal model
	In vivo
	rat
	mice
	murine
	Animals/not (Animals/and Humans/)
	(animal∗ OR in vivo OR preclinical)

### 2.2. Eligibility Criteria

Preclinical studies that induced cardiac injury through ischemia or ischemia–reperfusion, specifying the injury induction process and the perioperative activities performed on the study population, were included. Restrictions associated with the duration of injury induction, type of cell therapy, and tissue engineering strategy were not considered.

### 2.3. Data Selection Process

A database with 586 original articles in English was created from the review of titles and abstracts. Duplicate citations were removed. After making additional selections based on the methodology and results sections, only the articles with detailed information on the TD implantation process and the variables analyzed were included.

### 2.4. Information Analysis

**Figure 1:**
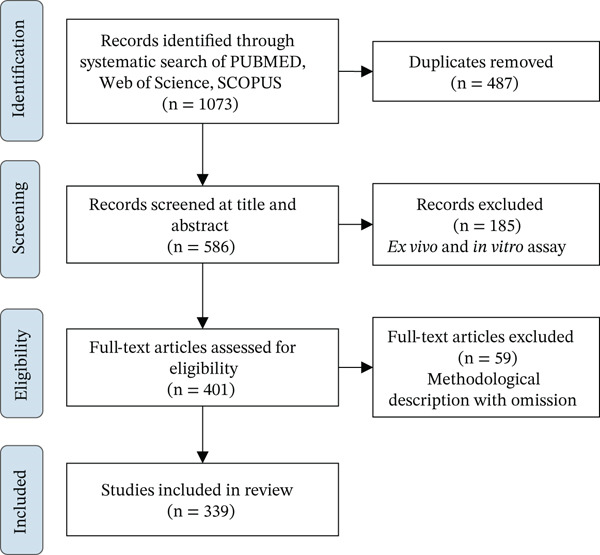
Process flowchart for the identification of sources of consultation.

The following characteristics of the 342 articles that met the inclusion criteria were compiled: publication details, animal model, TD, experimental design, and variables analyzed at the physiological level and histological level (Figure 1).

A first analysis describes the percentage of original publications based on the strategy of cardiac regeneration, and TD development trends validated in murine models. A second analysis describes the main characteristics of the experimental design reported for TD validation. Finally, the strategies that have reported the best values related to LVEF and infarct size of 51 articles reported as constructs are compared.

## 3. Results

A total of 1073 literature studies were identified in the last 10 years, and 586 were selected and read as full text after screening the titles and abstracts. Subsequently, 339 studies met our eligibility criteria and were included in this study for further qualitative analysis on trends in preclinical validation of cardiac regenerative therapies. Finally, 51 studies were used to analyze the preclinical benefits of generating three‐dimensional (3D) structures that permit both sustained and local release of factors to the heart.

Based on our research (Figure 2), preclinical validation has focused on the search for cellular sources (14%, *n* = 49), the design of novel scaffolds (41%, *n* = 140), the development of acellular strategies (1%, *n* = 5), and use of cell‐seeded scaffolds (constructs) (43%, *n* = 145).

Figure 2(a, b) Cardiac regenerative therapy approaches validated in murine models. Proportion of approaches reported from 10 years. Research has focused on the use of natural biomaterials for the incorporation of cells and other molecules. Tissue limitations can be addressed by incorporating biological factors to functionalize the therapeutic proposal.

**Figure:**
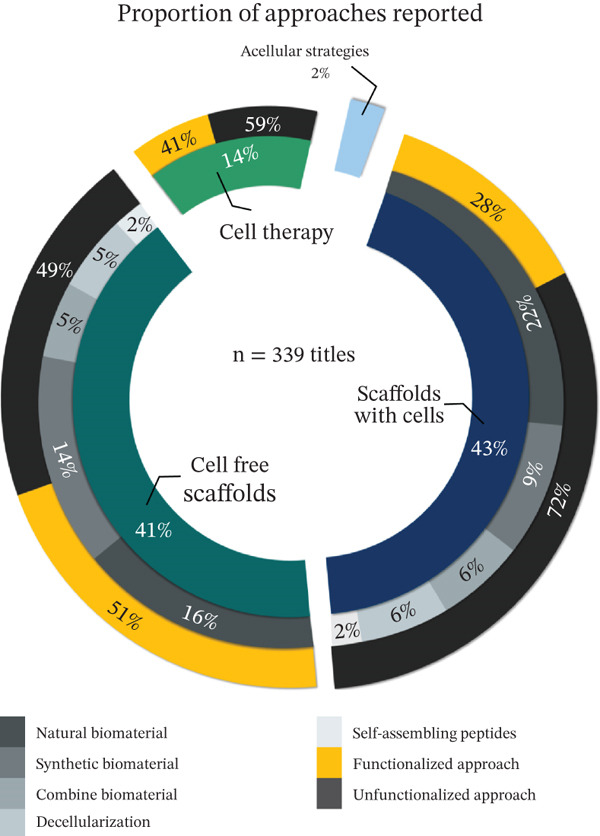
(a)

**Figure:**
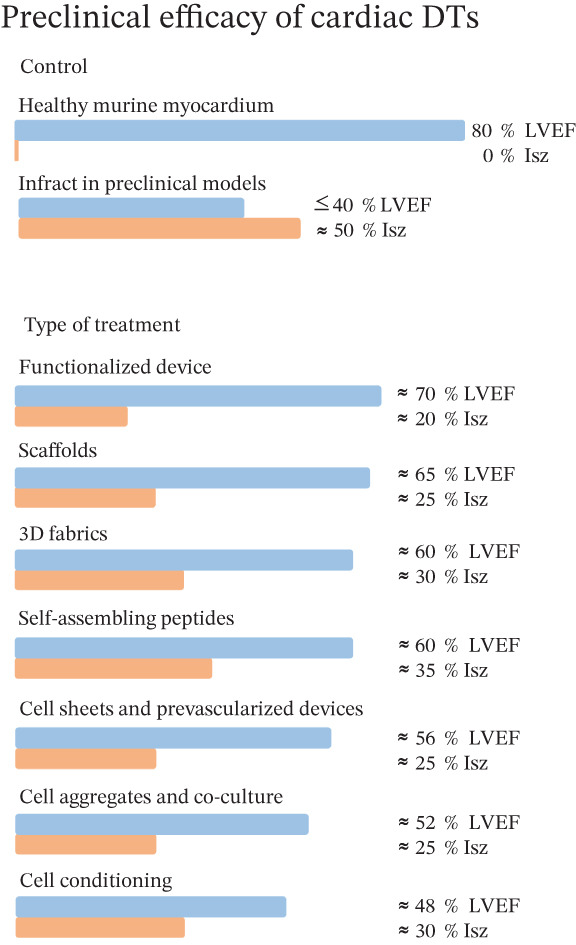
(b)

**Figure 3:**
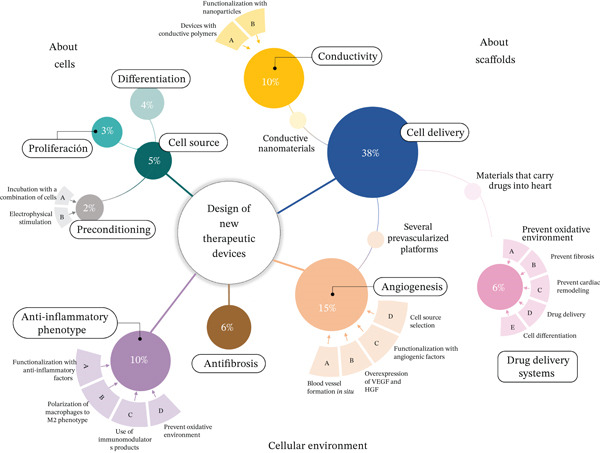
Trends in cardiac regenerative therapy. Priority areas that will allow scaling the cardiac tissue engineering strategy safely and efficiently to the clinical area. Preclinical validation has focused on the search for novel strategies for cell delivery to infarcted tissue, proper cell proliferation and differentiation, new cell sources, blood vessel formation, development of an anti‐inflammatory phenotype, and the search for electromechanical coupling of resident and implanted cardiomyocytes.

According to the objective of each screening title, trends in cardiac tissue engineering were established (Figure 3). The common objective, in the last 10 years, has been to develop a suitable cellular support (scaffold) that provides appropriate mechanical support to maintain structural stability in vivo and in vitro and, at the same time, ensure biodegradability and compatibility with cell and tissue regeneration. Recently, research efforts have focused on generating strategies to address the inflammatory environment after AMI, promote blood vessel formation, limit fibrosis, pursue cell proliferation in situ, and promote adequate conductivity between the tissue and the implant (Table 2).

**Table 2:** Key areas of progress in cardiac regenerative therapy.

Approach	Percentage of targeted articles	Aim	Description	Contributions
New cell‐based approaches	5%	To propose cell sources for the regeneration of infarcted myocardium.	Consider the clinical challenges of cardiac regenerative therapy to search for available alternatives.Search for different cell types easily obtained and rapidly cultured, with a high capacity for differentiation into cardiac cells with minimal atherogenic risk and greater anti‐inflammatory capacity.Special interest in the search for cell sources that promote vascular growth.	Mesenchymal cells derived from adipose tissue (ADSMC) [16, 17] and placenta (hAMSCs) as cell resources [18–20].
Cell differentiation	4%	To induce diverse cell types into cardiac cells.	It addresses the need to generate cardiac cells.Several strategies seek to reprogram stem cells into cardiomyocytes at the site of injury or in vitro, as well as to predifferentiate MSC to ensure cell availability, adhesion, and survival.These strategies include device functionalization with vector virus carrier molecules, in vitro treatments, and the search for new differentiation protocols.	Direct cardiac reprogramming [21, 22], gene delivery system [23, 24], and small molecules loaded into hydrogels [25, 26].
Cell survival and proliferation	3%	To prevent cell death by apoptosis and necrosis.	It is aimed at generating a large number of cardiac cells. It takes into account cell loss following the delivery of cell therapy as a consequence of the unfavorable environment in the infarcted tissue.Search for a microenvironment that allows cell proliferation through the addition of molecules for functionalization of the device and delivery of extracellular vesicles, with the aim of reducing oxidative stress in the infarcted myocardium, promoting the activation of MMPs, and promoting vascularization of the implanted tissue.	iPSC‐CM [7, 27], nongenetic approach to increase cell division [28], genetic modification of MSC [23, 29], and small molecules loaded into hydrogels [30–32].
Cell delivery	38%	To prevent cell death by anoikis.	This stems from low cell retention rates during cell therapy delivery.A variety of techniques are used to improve the adhesion of the device to the host tissue and prolong cell retention. The use of one or several support elements, the formation of structures with assembly properties in situ, or the prior formation of 3D tissues and cell aggregates are usually proposed.	3D construct design [33, 34], hydrogels polymerizable with UV light [35, 36], innovation in biomimetic systems [37, 38] and sutureless patches [39, 40], 3D bioprinting [41–43], self‐assembling peptides [44, 45], use of decellularized ECM [46, 47], and design of thin, porous and conductive scaffolds [16, 48, 49].
Blood vessel formation	15%	To promote blood vessel formation in the implanted device.	The lack of oxygen supply to the infarcted myocardium causes implanted tissues to depend on passive diffusion from the surrounding tissue. This results in poor viability and increased death of transplanted cells.	Overexpress VEGF protein by transfecting iPSC‐CMs with VEGF mRNA prior to transplantation [50–52]. Development of a prevascularized cardiac patch through various strategies including the use of cell aggregates, layer‐by‐layer culturing, micro engineering for the formation of a network of blood vessels in vitro, or through the use of cell‐seeded scaffolds that serve as templates for vessel formation [33, 48, 53–57]. Functionalization of the device by the addition of vascular endothelial growth factor (VEGF), hepatocyte growth factor (HGF), and fibroblast growth factor [58–60].
Development of an anti‐inflammatory phenotype	10%	To prevent cell death by pyroptosis.	The low retention and survival of implanted cells are attributed to hostile hyperinflammation in the ischemic region. Regulation of the inflammatory response at the site of injury is critical for tissue repair. Thus, research has focused on the search for macrophage polarization, clearance of excessive ROS, and the use of functionalized scaffold with antioxidants and anti‐inflammatory cytokines.	Macrophage immunotherapy, black phosphorus nanosheets, Ros‐cleavable polymers, hydrogen sulfide donor scaffold, curcumin, and anti‐inflammatory cytokines functionalized scaffolds [45, 61–67].
Myocardial fibrosis reversion	6%	To harness interactions between biomaterials and the extracellular matrix to elicit more native cellular phenotypes.	It takes into account the growing interest in targeting pathways that lead to altered cardiovascular cell phenotypes and microenvironments after injury to reduce maladaptive repair and promote functional recovery.Utilize the dynamic microenvironment of the cardiac scar with heterogeneous fibroblast populations. Send appropriate environmental cues so fibroblasts can help coordinate cardiac repair. By sending exosomes that alter fibroblasts’ activation, or through the sustained release of peptides to attenuate remodeling, and seek to minimize the degree of degradation of the cardiac ECM.	Attenuation of cardiac ECM degradation by matrix metalloproteinase inhibition [68, 69] and delivery of antifibrotic drugs or bioactive factors to inhibit specific signaling pathways related to fibrosis, for example, transforming growth factor‐*β* (TGF‐*β*) [70], miR‐29 [71], basic fibroblast growth factor (FGF‐2) [72], recombinant human ACE2 (rhACE2) [73], and bone morphogenetic protein 9 (BMP9) [74].
Cell preconditioning	2%	To promote cell survival by conditioning the cells.	Conditioning of the cell source for the inflammatory environment and the heartbeat. Conditioning is performed in vitro, through stimulation, coculture, and nutrient modification protocols in cell culture.	Electromechanics stimulation [75–78], coculture systems [79, 80], culture with cytoprotective molecules [81], and modification of oxygen availability during culture [82, 83].
Electrical communication in the myocardium	10%	To enhance the electrical conductivity in the implant.	It works on the lack of coordination between the implanted cardiomyocytes and the resident cardiomyocytes in the damaged tissue, which leads to the development of arrhythmias.	Development of devices with conductive polymers [9, 10, 16, 49, 84–86] or functionalization with molecules that improve the electrical conduction of the device [25, 87–91]. In addition, protocols for electrical stimulation of cardiomyocytes in culture are under development.
Sustained drug release	6%	To control drug release rate.	Sustained drug release systems prolong the retention of therapeutic drugs within target tissues to alleviate the need for repeated drug administration.The technology for developing nanodrugs, in functionalized scaffolds, allows the quantitative release of the drug to be delayed and the amount of medication needed to be reduced, improving the MF treatment strategy.	Nanocarriers based on lipids, peptides, hyperbranched polymers, microspheres, and covalent organic frameworks have been reported [92–102].Drugs such as pirfenidone and insulin‐like growth factor 1 (IGF‐1) as antifibrotic agents [98, 103]; colchicine, astragaloside IV, and IL10 as anti‐inflammatory agents [96, 97, 104]; and dapagliflozin, tanshinone IIA (TIIA), and irbesartan as infarct treatment agents [101, 105, 106] have been used.

**Figure 4:**
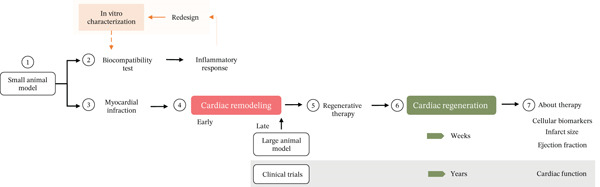
Process flowchart for preclinical validation of cardiac regenerative therapy. Once the TD has been characterized in vitro, preclinical murine validation should consider and report within the experimental design: (1) the characteristics of the animal model, (2) the biocompatibility analysis of the biological environment in response to the material—an analysis prior to development of the infarction, (3) the type and (4) time of lesion progression, (5) the choice and delivery route of the TD, (6) the time of cardiac regeneration, and (7) the analysis of the variables in response to treatment.

A homogeneous preclinical scenario is required for the validation of TDs. From the reported methodology of each screening titles and from our perspective, the experimental design to validate a TD in murine models requires seven key moments (Figure 4) to generate a complete and adequate results report, which allows the identification of therapies that promote the best results based on the restoration of cardiac function and guides the design of new therapies and their application in clinic.

### 3.1. Experimental Design Reported for Preclinical Validation of Cardiac Regenerative Therapy

This paper lists key points to consider for when choosing the murine model and the method of AMI induction, as well as relevant aspects on the timing of implantation and evaluation of therapy.

#### 3.1.1. Stage 1: Murine Model

Rats were used in 74% (*n* = 232) of papers, in particular, the Sprague–Dawley strain (64%, *n* = 138). The AMI induction process could explain this trend, as the size of the heart could affect the outcome of the surgery, making tissue handling difficult in mice. Among the least represented strains of rats and mice are Wistar (20%, *n* = 42) and Lewis (7%, *n* = 15). Males were used in 75% (*n* = 175) of papers. The low representativeness of females (25%, *n* = 58) represents an opportunity that may contribute to the scope of the device since estrogen is involved in left ventricular remodeling [107]. The activation of estrogen receptors (ERs) *α* and *β* promotes the expression of antioxidant enzymes (SOD‐2) and proteins involved in the control of vascular tone (eNOS and COX‐2); in addition, it downregulates the expression of proinflammatory (TNF‐*α*) and profibrotic (TGF‐*β*1) [108, 109] cytokines. ER activation in the cardiovascular system could increase the survival rate of the cells implanted in the TD by promoting an anti‐inflammatory phenotype. Although AMI is more common in men, women have a higher risk of dying after a heart attack [110]. Women often present atypical symptoms (neck, jaw, shoulder, or back pain, unusual fatigue, shortness of breath, etc.), which can delay diagnosis and treatment [108]. Factors such as smoking, obesity, stress, and sedentary lifestyle outweigh the protective benefits of female hormones, leading to a gradual increase in the incidence of AMI in young women [111]. Therefore, insight into the role of the ERs could be useful for the development of new cardiac TDs.

#### 3.1.2. Stage 2: Biocompatibility Analysis

To validate cell‐free and cell‐seeded scaffolds, a biocompatibility test should be performed prior to AMI induction. Biocompatibility tests are a first step in the preclinical evaluation of a cardiac TD. The implantation of any biomaterial elicits a local or systemic response from the host tissue. This response is termed as a foreign body response, and its mechanism has been extensively revised [112]. In brief, after implantation, all foreign materials are surrounded by collagen deposition in the form of a fibrotic scar at the material‐tissue interface. This interface must support cellular activity without hindering the signaling cascade in response to implantation. Since the intensity of the inflammatory response is mainly determined by the composition of the biomaterial, porosity, hydrophobicity, topography, and biodegradability of the scaffold, or even by any of the components (functionalization molecules) of the TD [113, 114], it is important to identify the recruitment and reactivity of cellular mediators after implantation.

The goal of biological response evaluation is to predict whether an implant presents potential harm by evaluating its performance in conditions that simulate clinical use. The design for biocompatibility analysis includes subcutaneous implantation of the TD for 1–4 weeks in the dorsum or chest of the animal. These tests are used to explore the local and systemic inflammatory response triggered by the implantation, the integration between host cells and the construct, the number of new vessels, the expression of biomarkers, and the rate of degradation [115, 116]. In addition, cell death and differentiation are analyzed during the validation of a cell‐seeded scaffold [117]. Observations are made considering the phases of acute inflammation (3 days or less) and the resolution of the inflammatory response. Observations usually tend to 7, 15, and 28 days after implantation. Biocompatibility testing enables the analysis of the effects of foreign agent implantation and interactions with surrounding cellular elements, as well as the identification of the recruited immune system cells [115, 116].

#### 3.1.3. Stage 3: AMI Induction

The most commonly reported procedure to induce AMI is coronary occlusion (96%, *n* = 325). Other procedures include chemical, mechanical, and cryoinjury. Although AMI can be induced by any of the previously mentioned procedures (Table 3), the type and mechanism of injury and the extent of reproducibility among animals differ in each case. As a result, remodeling events and the infarct size are different.

**Table 3:** Reported AMI induction models for the validation of cardiac regenerative therapy.

**Induction**		**Model**	**Intervention**	**Model preparation**	**Induction procedure**	**Type of event**	**Type of injury**	**Mechanism of injury**	**Determinant of injury size**
Ischemia		Permanent LAD occlusion	Surgical. LAD Identification	Advances in pre‐ and postsurgical animal care include the use of analgesia, anesthetics, and asepsis for the care of the specimens and to improve the scientific reliability of the results:1. Most used analgesics: buprenorphine, carprofen, and bupivacaine.2. Most used anesthetics: ketamine, xylazine, and isoflurane.3. Tracheotomy. Endotracheal intubation necessary to control artificial ventilation and administration of anesthetic to the specimen.4. Thoracotomy. Incision in the left fourth intercostal space.	Secure suture around the LAD. Confirmation of the injury by myocardial blanching or ST‐segment elevation on echocardiography.	Chronic	Coagulative necrosis	Lack of oxygen causes mitochondrial damage, increased Ca^2+^, and cell death.	Fixed infarct area 24 h after ligature placement
Ischemia/reperfusion		Transient LAD occlusion (20–60 min)	Surgical. LAD identification		Secure suture around the LAD. Allow reperfusion and confirm through myocardial color change or ST‐segment change on echocardiography.	Acute	Apoptosis and localized portions of coagulative necrosis	Following ischemia damage, oxygen restoration leads to oxidative stress damage.	Ischemia duration
Cryoinjury		Liquid nitrogen application	Surgical		Precool the probe. Apply the probe to the anterior wall of the left ventricle and freeze for 10 s.	Acute	Necrosis	Crystal formation and disruption of the cell membrane.	Probe size
Ablation		Cardiac biopsy	Surgical. Generation of a “bulge” in the myocardium by means of a tourniquet		The bulging area is excised almost entirely to create a defect ~2–3 mm thick.	Acute	Necrosis in the transition zone	Mechanical damage. Loss of cardiomyocytes due to tissue removal.	Tourniquet diameter
Chemical		Isoproterenol injection, *β*‐adrenergic receptor agonist	Pharmacologic. SC or IP admin. of isoproterenol for 2 days every 24 h	Not necessary	Generation of AMI with doses above 100 *μ*g/kg, lower doses cause reversible damage.	Chronic	Apoptosis	Mitochondrial dysfunction, changes in the concentration of fatty acids, and Ca^2+^. Production of free radicals.	Dose, time of administration

**Induction**	**Histological detection of injury^a^**	**Molecular markers^b^**	**Mortality**	**Injury size**	**Advantages**	**Disadvantages**	**Information reported**	**Number of articles**	**References**
Ischemia	H&E, TTC, Masson’s trichrome, Sirius red	Cell damage: AKT‐1, Wnt‐3, cleaved caspase‐3, TLR‐4.Oxidative damage: peroxidase, SOD.Myocardial damage: troponin T, troponin I.Inflammatory process mediators: Il6, Il1*β*, Cxcl10, TNF‐*α*, Il6, Il1*β*, Cd14, Mertk, CD68, MMP2, MMP9.Cardiomyocyte regeneration: NRG‐1, H3K4 me3, ErbB2, 3, 4.Neovascularization: CD31.Fibrosis: p‐SMAD2, Tenascin‐C, Tgfb, Ctgf, Postrn, Il11.	≤ 40%	≤ 60% LVA	Robust remodeling response; large injury size.	The injury does not represent the reperfusion damage present in the clinical situation.	Suture size and location of occlusion.	282 (83%)	[118]
Ischemia/reperfusion	H&E, TTC, Masson’s trichrome, Sirius red, phthalo blue		≤ 40%	≤ 30% LVA	High representativeness of the clinical mechanism of AMI.	High mortality rate associated with surgery, which decreases after the learning curve of each laboratory.	Ischemia and reperfusion duration, suture size, and location of occlusion.	41 (12%)	[119]
Cryoinjury	H&E, TTC, Masson’s trichrome		< 20%	15%–30% LVA	Reproducibility of injury size and location; complete loss of cardiomyocytes.	Low representativeness of the clinical mechanism of AMI.	Type of probe or tube; location, duration, time, and number of lesions.	8 (2%)	[120]
Ablation	H&E, TTC, Masson’s trichrome		Not recorded	≤ 80% LVEF	Reproducibility of injury size and location; complete loss of cardiomyocytes; infarction size and location independent of coronary anatomy.	Low representativeness of the clinical mechanism of AMI. Injury generated from the epicardial surface to the myocardium.	Suture size, ablation size.	3 (1%)	[121]
Chemical	H&E, Gomori, Masson’s trichrome, Sirius red		~25%	20% LVA	Low mortality of the animal model; rapid induction without requiring surgery.	Low control of lesion transmurality. Injury areas located in the left ventricle, septum, papillary muscle, and apex of the heart.	Dose, time, and route of administration.	5 (2%)	[122]

Abbreviations: H&E = hematoxylin and eosin, IP = intraperitoneal, LVA = left ventricular area, LVEF = left ventricular ejection fraction, SC = subcutaneous, TTC = tetrazolium chloride.

^**a**^Most reported stains for the identification of AMI in murine models.

^**b**^Most reported molecular markers in induction in murine models.

Coronary occlusion–induced AMI requires surgical intervention on the animal to ligate the left anterior descending (LAD) coronary artery. These surgical models can lead to chronic cardiac ischemia (occlusion by permanent ligature) or ischemia/reperfusion injury (occlusion by transient ligature). In both cases, occlusion causes immediate arrest of the myocardial aerobic metabolism [123]. In chemical induction using isoproterenol (ISO), oxygen deprivation originates from myocardial hyperfunction [122]. ISO is a *β*‐adrenergic receptor agonist that leads to increased chronotropism, inotropism, and hypotension in the coronary circulation, which results in an imbalance between oxygen supply and demand by cardiomyocytes (CMs) [124].

Cryoinjury is produced by a freezing process that increases the volume of aqueous solutions and generates mechanical forces that damage the cell membrane. The application of liquid nitrogen onto the myocardial wall causes an irreversible injury [120]. Ablation is the direct removal of cardiac tissue by surgery [121]. Coronary occlusion, ablation, and cryoinjury facilitate the precise timing, location, and extent of the coronary event, leading to highly reproducible results [13]. However, chemical induction leads to a wide injury area beyond the left ventricular myocardium. Therefore, the permanent and transient occlusion models are representative of subendocardial AMI, and the chemical, cryoinjury, and ablation models represent the transmural infarction condition.

AMI induction in the animal model provides a basis for assessing the efficacy of the TD. Therefore, problems associated with reproducibility and bias in the development of the injury should be minimized. Permanent LAD occlusion was reported in 84% of the reviewed articles. This model represents ischemia damage, which generates an infarct size of ≤ 60% of the left ventricular area [118]. In clinical practice, reperfusion coupled with the administration of other therapies is part of the primary care of infarcted patients [125]. This reduces in‐hospital mortality within the first 12 h from the onset of symptoms [126]. Given that the pathophysiology and final injury size are affected by reperfusion, the permanent occlusion model does not represent the scenario described for patients with AMI. Nevertheless, this model could be used for chronic cardiac ischemia events. The transient occlusion model was reported only in 12% of studies on cardiac TD preclinical validation and involves ischemia and reperfusion damage. Although 49% of cases using transient occlusion report 30 min of ischemia [5], a 30% lesion of the ventricular area is generated in only 20 min. Considering that both strategies include an invasive surgical procedure, the use of the transient occlusion model is recommended in the experimental design for the validation of cardiac TDs because it is more similar to the clinical scenario.

#### 3.1.4. Stage 4: Cardiac Remodeling and TD Implantation

The analysis of the efficacy of TDs is complex, especially when considering parameters such as time of implantation, cardiac biomechanics, and oxygen supply to the injured tissue, which affect cell adhesion and survival. Hence, after AMI, it is important to define the time of intervention.

For studies about early remodeling, therapy should be delivered ≤ 72 h after the ischemia. Among the reviewed articles, 77% (*n* = 260) delivered when the AMI occurs, or a few minutes after myocardial bleaching (AMI confirmation in surgery). These protocols evaluate processes associated with expansion of the injury area and changes in extracellular matrix (ECM) structure [127]. Late cardiac remodeling was evaluated in 21% (*n* = 72) of the studies, when the intervention is longer than 7 days after the ischemia. At this time, ventricular dilatation, cardiac hypertrophy, and scar thinning are analyzed [123, 128]. Generally, studies with a late remodeling scheme perform a second thoracotomy, which impacts the survival rate. Finally, only 2% (*n* = 7) of studies compare the effect of therapy at both times.

The scar tissue undergoes a maturation process in which the cellular and ECM is cross‐linked and the repairing cells undergo apoptosis. The TD efficacy may diminish in stages consistent with the acute inflammatory process or after scar formation. In this regard, studies on late implantation showed limited improvement in LVEF compared to early implantation [129]. After infarction, scarring involves a variety of inflammatory cells and the activation of matrix metalloproteinases (MMPs). MMPs degrade ECM material, assisting macrophages in the reabsorption of necrotic tissue. A 50% loss of ECM collagen has been estimated within the first 3 h after ischemia. This inflammatory scenario could be associated with a low cell survival rate [130]. According to our analysis, 75% of the studies implanted the TD during the inflammatory phase, 17% during fibroblast proliferation, and 8% during scarring. The experimental design of the preclinical studies that implanted the TD during the late remodeling phase included coupling with other therapies to revert ventricular remodeling, such as surgical ventricular restoration [131], bypass implantation, and ventricular assist device [132, 133]. Coupling therapies could be advantageous at a time when wall thinning and the dense nature of scar tissue hinder single cardiac TD implantation.

Despite enormous efforts to accurately represent the clinical scenario, the survival rate of the murine model after surgical induction limits the implantation of the therapy to early stages of chronic occlusive injury development. Clinical trials on cardiac regenerative medicine include patients as early as 3 weeks after hospital admission for myocardial infarction, which corresponds to an advanced stage of cardiac remodeling [134–136]. Surgery to induce AMI in rats reflects the damage observed in infarcted patients participating in clinical trials, even though the progression of the injury is driven by different events.

A common obstacle in surgery is the delivery of the TD. Delivery routes include injection of hydrogels into different sites of the ventricle (68%, *n* = 213), which implies the proper use of the technique to avoid administering the therapy inside the ventricle, suture a sponge to the ventricle (25%, *n* = 80), and the use of fibrin glue (5%, *n* = 15). Minimally invasive surgical techniques that allow for early cell delivery during the acute AMI phase are under exploration to prevent hydrogel dissemination at the implantation site and avoid causing damage to cardiac tissue. Recently, the use of structures polymerizable with UV light has been reported (2%, *n* = 5) [35, 36, 137, 138], where the rapid polymerization of a hydrogel can take the shape of the target tissue. Another alternative is the sutureless TD design as reported [39, 87].

#### 3.1.5. Stage 5: Regenerative Therapy

The wide array of tissue engineering approaches includes the use cell and hydrogel injection (56%, *n* = 190) [139, 140], 3D constructs (28%, *n* = 95) [33, 34, 141], decellularized ECM as a scaffold (11%, *n* = 37) [142–144], self‐assembly molecules (4%, *n* = 12) [8, 145, 146], and 3D bioprinting (1%, *n* = 5) [41, 147], as the main strategies reported (Table 4). When one or more molecules are added to the TD to generate a favorable microenvironment for the implanted cells, the strategies are called “functionalized therapies” (40%, *n* = 135) [25]. Functionalization enhances the benefits of TD therapy. However, if only the cell therapy and the biomaterial are included, they are called “nonfunctionalized therapies” (60%, *n* = 204) that represent the most reported strategy.

**Table 4:** Strategies reported for preclinical validation of cardiac regenerative therapy.

Strategy	Description	Advantages	Efficacy		Limiting factors	References
			Infarction (%↓)^a^	LVEF (%↑)^a^		
Cell sheets	Biomembrane of cells that are placed on the surface of the heart.	Improvement in the initial retention of implanted cells.	≤ 50	≤ 40	Oxygen supply. Efficacy associated with the “thickness” of the sheet.	[148, 149]
Coculture	Stem cell culture with cardiomyocytes, or with other cell types.	Improvement in cellular communication due to increased expression of connexin 43.	≤ 50	≤ 20	Previous culture time of the different cell types.	[36, 150, 151]
Cell preconditioning	Culture under hypoxic conditions and electrophysical stimulation.	Increased cell proliferation and proper differentiation into different cardiac cell types.	≤ 40	≤ 20		[76, 82, 83]
Cell aggregates	Combination of cell therapy. Deliberately prespecified designs with multiple functions intended to improve therapeutic efficacy.	Mitigation of challenges of single‐cell delivery through spherical self‐assembly. Promotion of cell survival, differentiation, and proliferation.	≤ 50	≤ 30		[152–154]
3D tissues	Formation of 3D multicellular tissues of suitable size for entrapment in the muscle interstitium.	Improved cell retention and supply of oxygen and nutrients during culture.	≤ 40	≤ 50	Long‐term cell survival associated with the absence of new blood vessel formation at the site of injury.	[33, 34, 56]
Prevascularized devices	Formation of a vascular network in culture from cell‐only or scaffold‐supported approaches.	Improved supply of nutrients and oxygen across the tissue after implantation.	≤ 50	≤ 40	Retention in the infarcted area.	[53, 55]
Scaffolds	Tissue‐engineered constructs with biomaterials of different nature (natural, synthetic, or combined), which act as a support for cell growth and differentiation.	It accepts different cell types and allows the establishment of interactions similar to those in native tissue. It allows the establishment of a vascular network and functionalization of the device.	≤ 60	≤ 50	Specific complications related to biocompatibility, degradation rate, and mechanical resistance to heart beating.	[9, 151, 155, 156]
Bioprinting	Creation of cardiac patches, scaffold‐free or not, by multicellular assembly using a 3D bioprinter.	Uniform patch thickness, precise pore size, and structure formation, mimicking the outer and inner architecture of native tissues.	≤ 30	≤ 50	Failure in coordination between the host and implanted tissues.	[41, 141, 147]
Self‐assembly peptides	Polypeptides that under certain physiological conditions are capable of assembling into 3D scaffolds that resemble the extracellular matrix.	Support structures for the growth and differentiation of various cell types. Their degradation products are easily absorbed.	≤ 30	≤ 50	Long‐term cell survival (> 4 weeks).	[8, 44, 146]
Small extracellular vesicles	Delivery of stem cell–derived extracellular vesicles containing multiple soluble proteins, mitochondrial DNA, mRNA, and noncoding RNA.	Reduction of cell death. Protection of cardiomyocytes against apoptosis.	≤ 60	≤ 40	Retention in the infarcted area. Short half‐life.	[27, 145]
Device functionalization	Addition of organic or inorganic molecules to the TD.	Promotion of damaged tissue remodeling (cytokines, chemokines), blood vessel formation (VEGF, NGR1, EPO, and GE), electrical conduction and coupling of new cardiomyocytes to the host tissue (carbon nanotubes, AuNP, and Ti), and reduction of oxidative stress (IGF‐1 and Gr).	≤ 60	≤ 60	Retention in the infarcted area. Short half‐life.	[5, 28, 69, 131, 157, 158]

*Note:* Molecules usually added to functionalized device.

Abbreviations: EPO = erythropoietin, GE = genistein, GE = graphene, IGF‐1 = insulin‐like growth factor 1, NGR1 = neuregulin, AuNP = gold nanoparticles, Ti = titanium, VEGF = vascular endothelial growth factor.

^**a**^Measurements recorded at the end of the protocol, comparisons with respect to the infarcted group.

The strategies used for cardiac regeneration have focused on promoting an adequate cellular environment. This has been achieved through functional proposals. Therefore, the manufacture of cardiac patches could be the option that brings together specific benefits to achieve the success of cardiac regenerative therapy. The analysis of the approaches that have been addressed in cardiac regenerative medicine is described in Table 2. Our analysis has identified 10 priority areas related to cell source, cell differentiation and proliferation, cell delivery, and TDs proposal to reduce inflammation and fibrosis after AMI, promote blood vessel development, and ensure electrical conductivity between native tissue and implant, TD preconditioning, and drug delivery systems, all aimed at developing new TDs. Based on bibliographic evidence, historically, the search to improve cellular delivery has been replaced by generating complex structures that include more than one topic listed above to ensure the damaged tissue recovery. The findings found in each area are described below (Figure 3).

Cell therapy research focuses on the search for one or more optimal cell sources for cardiac regeneration. Most reported cell types are listed in Table 5. The use of mesenchymal cells (MSCs) has been mostly reported (51%, *n* = 101). Among such cells, bone marrow mesenchymal stem cells (BMSCs) (32%, *n* = 33) are known to have multiple paracrine effects for cardiac repair such as angiogenesis, anti‐inflammation, antiapoptosis, and immune modulation [34, 39, 172]. Historically, this cell source has been used in tissue engineering. Recently, evidence reports that adipose‐derived stem cells (ADSCs) (40% of MSC reports) represent a better alternative. It has been documented that ADSCs have a greater capacity to proliferate towards CMs and can be obtained easily from subcutaneous fat via minimally invasive procedures [16, 17, 173]. Although some reports from animal studies have revealed the abilities of MSCs to repair damaged tissues, a successful stem cell therapy method for injured cardiac tissue regeneration has not reached the clinical stage yet. In this sense, human amniotic membrane mesenchymal stem cells (hAMSCs) have been proposed (11%, *n* = 11); hAMSCs positively affect the repair of myocardial ischemia–reperfusion (MI/R) injury by stimulating endogenous repair mechanisms [174–176]. In addition, the use of exosomes derived from MSC has been reported (16%, *n* = 16) as a cell‐free approach. They are considered promising carriers for therapeutic agents due to their intrinsic biocompatibility, low immunogenicity, and ability to cross physiological barriers [177]. Also, they can protect the encapsulated materials from degradation, thereby enhancing therapeutic efficiency [46].

**Table 5:** Cell sources reported for preclinical validation of cardiac regenerative therapy.

Cell source	Reservoir	Cell potency	Applications	Limiting factors	Number of articles	References
Cardiac progenitor cells	Atrium and ventricular apex	Multipotent	Resource of cardiomyocytes, endothelial, and smooth muscle cells.	Ethical issues in the translation to the clinical model.	9 (5%)	[8, 41, 159]
Cardiac stromal cells	Atrium and ventricular apex	Multipotent	Resource of cardiomyocytes, endothelial, and smooth muscle cells.	Low cell density.	9 (5%)	[53, 142, 160]
Neonatal cardiac cells	Ventricle	Differentiated	Resource of cardiomyocytes. Used in coculture to induce spontaneous rhythmicity.	Blood vessel formation.	15 (8%)	[9, 77, 91]
Embryonic stem cells	Cell line	Pluripotent	Unlimited resource of contractile cardiomyocytes.	Ethical issues in the translation to the clinical model. Lack of electrophysical coupling with host tissue. Development of arrhythmias and teratomas.	6 (3%)	[19, 161–163]
Umbilical cord stem cells	Umbilical cord vein endothelial and cord blood mesenchymal cells	Multipotent	Resource of endothelial and smooth muscle cells. Promotion of angiogenesis.	Decreased host immune response. Long‐term blood vessel formation. Procurement of contractile cardiomyocytes.	11 (6%)	[20, 26, 146, 151]
Endothelial progenitor cells	Bone marrow from long bones and peripheral blood	Multipotent	Resource of endothelial cells. Repair of endothelial function. Neovascularization.	Low cell retention rate.	10 (5%)	[164–167]
Muscle progenitor cells	Skeletal muscle	Unipotent	Approach for rapid isolation and high availability. Neovascularization. Ischemia resistant resource.	Low cell retention rate.	4 (2%)	[168, 169]
Mesenchymal cells	Cell line, bone marrow, and adipose tissue	Multipotent	Cardiac cell reservoir. Approach of low immune response. Promotion of paracrine effects. Neovascularization.	Low cell retention rate. Cell survival. Culture time.	101 (51%)	[143, 144, 155, 156, 170]
Induced pluripotent cells	Induced from cell types other than cardiomyocytes	Pluripotent	Unlimited resource of cardiomyocytes.	Adequate differentiation and immune rejection. Identification of teratomas.	33 (17%)	[7, 28, 171]

In order to generate abundant cardiac cells, a vast number of cell proliferation and differentiation protocols have been reported. The use of human induced pluripotent stem cell (iPSC)–derived CMs is common among publications (17%, *n* = 33). Greater cell proliferation has been documented when using iPSC. The efficiency of cardiogenic differentiation of CM‐derived iPSC was 53%–57% compared with 33%–39% for mouse embryonic stem cells and 25%–33% for cardiac fibroblast–derived iPSC [50]. Nongenetic approach to promote hiPSC‐CM cell cycle activity and proliferation in transplanted human cardiomyocyte patches (hCMPs) has been documented in [28]. However, there are many hurdles to the clinical application of hPSC therapy. One of the major obstacles is the potential immune rejection of hPSC‐CMs posttransplantation. Given this, it has been proposed the use of human amniotic fluid‐derived stem cells (hAFSCs) that are immunologically privileged but cannot induce cardiac differentiation. Therefore, it has been proposed to differentiate hAFSCs into iPSC [7]. Likewise, the use of immunosuppressants to test the effectiveness of induced cells (hiPSC) avoids rejection or differentiation towards another phenotype has been proposed [178].

Another approach reported to increase cell proliferation has been the genetic modification of MSC, direct cardiac reprogramming, and TD functionalized. In [23, 29], proliferation and survival in the hypoxic environment of the infarction are sought through the genetic modification of MSC for the expression of the hepatocyte growth factor (HGF) gene, a pleiotropic cytokine involved in cell regeneration. A promising genetic approach for both cardiac regeneration and antifibrotic therapy is direct cell differentiation in the tissue. The induction of CM in vivo has been reported in [21, 24], through the direct conversion of fibroblasts into CMs using cationic gold nanoparticles (AuNPs) loaded with various genes, or through the use of nonviral sequential targeting nanoparticles [22]. Finally, small molecules have been incorporated into hydrogel scaffolds to induce stem cell differentiation, such as 5‐azacitidine (5‐Aza) [25], inhibitor Wnt production‐4 [179], WIKI4 [172], and 2 ^′^‐deoxycytidine [26], to promote CM growth through functionalization with insulin‐like growth factor (IGF)–1 [30] and to protect CM against hypoxia and apoptosis through HIF‐1*α* encapsulated in MSC‐derived exosome [31] and NRG‐1 [61, 157, 180].

Different strategies aimed at improving biological capabilities and retention of cell sources include the combination of multiple cells, induction of cell aggregates formed ex vivo, and stem cell preconditioning in culture [82, 150, 152]. These strategies mitigate CM death, particularly under stress conditions, compared to implanted single‐cell types. Usually, iPSC‐CMs are cultured with one or several cell sources such as human umbilical cord–derived MSCs, ADSCs, and endothelial cells. This promotes cell–cell interaction, inducing a shift in cell phenotype towards a cardiomyogenic lineage due to more efficient transduction of molecular signals [83]. Likewise, CardioClusters [152], well‐defined cardiac cell populations including MSCs, EPCs, and CPCs, are also used. These approaches take advantage of the different phenotypic attributes of the cells used. Recent studies have also reported the use of cell aggregates, cardiospheres, and spheroids [147, 153, 154].

Cell retention and survival remain poor for both single‐cell and combined‐cell administration strategies [181]. In addition, transplanting multiple cell populations does not ensure effective cell–cell interaction [152]. Therefore, there is great interest in strategic approaches that seek to prolong cell retention and survival, preferably by favoring the phenotypic characteristics of cells that promote short‐term mitigation of injury [161] and long‐term recovery of myocardial structure and function [171]. Most efforts have focused on the search for correct delivery of cells into host tissue using various natural and synthetic scaffolds and, less frequently, scaffold‐free approaches. Increased cell retention has been reported with the addition of one of these strategies as a support for the cell source. Reported retention values were 10%–30% for cell‐only administration [182, 183] and 50%–80% for the joint administration of a cell source and a scaffold [23, 28, 155, 184]. A reduction of ≤ 60% in the infarct size of the infarcted myocardium was reported using these approaches.

The efficacy of cell therapy and tissue regeneration is closely related to the properties of biomaterials. The development of scaffolds requires biomaterials with adequate mechanical characteristics, which are biocompatible, biodegradable, and harmless to the host tissue [3]. In accordance with our analysis, most studies included natural biomaterials (44%, *n* = 124), such as chitosan (15%), alginate (15%), collagen (13%), and hyaluronic acid (13%), among others. Synthetic biomaterials have been reported in 26% (*n* = 74) of papers. The synthetic biomaterials most commonly used in cardiac tissue engineering, according to our analysis, are polietilenglicol (PEG) (29%), poly(caprolactone) (PCL) (29%), poly(lactic‐co‐glycolic acid) (PGLA) (27%), and poly(glycerol sebacate) (8%), among others. Synthetic biomaterials are an alternative for solving biomechanical problems; however, they face biocompatibility and degradability issues. Therefore, the combination of synthetic and natural biomaterials has been proposed in 13% of papers (*n* = 38). Sixty percent of the articles published since 2013 used a combination of biomaterials to create a complex scaffold [33, 37, 38, 185]. Other reported strategies were the use of decellularized ECM (13%, *n* = 37), mainly derived from myocardium, and self‐assembling peptides as scaffolds (4%, *n* = 12). A leading scaffold‐free approach uses self‐assembly peptides, which have high biocompatibility and the ability to incorporate various bioactive components [8, 44, 146]. For example, hyperbranched polymers are used as a novel strategy to minimize oxidative stress damage [145].

After AMI, innate immunity is rapidly activated to produce a strong and transient inflammatory response. Cell retention extending beyond the necrosis and inflammation phases remains a major challenge for cell therapy. In the analysis of our database, five strategies were identified that have been proposed to promote an anti‐inflammatory environment. First, the polarization of macrophages towards the M2 phenotype is sought. This has been achieved through the use of biomaterials that interact with monocytes [61, 186], by inhibiting and blocking glycolysis and suppressing the production of inflammatory cytokines [62], as well as by immunomodulation via the RGD interactions at the ECM [45]. Additionally, the use of amniotic products has been proposed as potent immunomodulators used clinically to repair tissue injury [63, 144, 187]. Purified human amniotic fluid is a nonantigenic solution of hundreds of naturally derived proteins that retain their anti‐inflammatory activity and can be used as a vehicle for cell maintenance after a heart attack. After AMI, excessive ROS is overproduced and accumulated in the myocardial microenvironment, which could not only induce strong inflammatory reactions but also observably restrict angiogenesis and lead to endothelial dysfunction. Due to the above, DTs have been generated for the delivery of antioxidants [64, 188, 189] and reactive oxygen species–scavenging scaffolds [65, 190–192]. Finally, strategies functionalized with molecules such as curcumin [46, 193], IL‐10, and 4/6/13 [5, 66], among others [67, 194, 195] have been reported to alleviate inflammation.

Following injury, cardiac fibrosis forms in the myocardium which greatly hinders cellular function, survival, and recruitment, thus severely limits tissue regeneration. In recent years, there has been growing interest in targeting pathways that lead to altered cardiovascular cell phenotypes and microenvironments after injury to reduce maladaptive repair and promote functional recovery. The approaches for preventing cardiac fibrosis after AMI generally involve the use of ECM elements to attenuate cardiac fibrosis [196, 197], attenuation of cardiac ECM degradation [68, 69], and delivery of antifibrotic drugs or bioactive factors to inhibit specific signaling pathways related to fibrosis, for example, transforming growth factor‐*β* (TGF‐*β*) [70], miR‐29 [71], basic fibroblast growth factor (FGF‐2) [72], recombinant human ACE2 (rhACE2) [73], and bone morphogenetic protein 9 (BMP9) [74].

Adequate oxygen supply represents another limiting factor for cell survival. To promote blood vessel formation, research has focused on the induction of blood vessel expression genes in transfected cells [50–52], through the combination of various cell sources, or through the design and functionalization of scaffolds. HUVECs, vascular progenitor cells, and cord blood‐derived endothelial colony‐forming cells have been reported as cell sources for angiogenesis [198–201]. More than 40% of studies focused on the development of angiogenesis have reported adding vascular endothelial growth factor (VEGF) or HGF to scaffold [58–60, 202]; other functionalization molecules that have been reported for blood vessel development are strontium ions [203], platelet‐derived growth factor (PDGF) [204], and angiopoietin‐1 (ANG‐1) [205]. A diverse series of strategies using scaffolds has been proposed. Biomaterials with angiogenic properties, such as alginate [206–208] and cardiac aorta‐derived ECM [209], among others, have been used to induce blood vessel formation in situ. Prevascularized cardiac patches are the most frequently reported strategies [33, 53–55]. Finally, novel TDs to promote microvasculation have been achieved with magnetic patch as to attract superparamagnetic iron oxide labelled endothelial cells [48], ultrasound‐controlled nano oxygen carriers in 3D GelMA hydrogel [56], and a photosynthetic oxygen delivery system through encapsulated *Synechococcus elongatus* into alginate hydrogel microparticles [57].

The foremost challenge in the restoration of the myocardial infarction is the increasing electrical behaviors of the implanted TD for electrical coupling between the cells and scaffolds. This problem can be overcome by the distribution of electrically and biologically active scaffolds. A significant number of conductive patches and injectable hydrogels have been developed to improve the coupling of graft and tissue. These have been synthesized with poly‐3‐amino‐4‐methoxybenzoic acid (PAMB) [9], carbon nanotubes (CNTs) [16, 210], polypyrrole (PPy) [40, 49, 84], hydrogen sulfide (H_2_S) [10], tetraaniline‐polyethylene glycol diacrylate (TA‐PEG) [85], and graphene oxide (GO) [86, 170]. The functionalization with nanoparticles is a usually reported strategy. The synthesized AuNPs have a biocompatible nature and also a high potential to improve intercellular electrical communications with cardiac cells, so functionalization with AuNPs has been widely used (64% of papers focused on scaffold conductivity through functionalization) [25, 87–89]. Other less reported nanoparticles were selenio [90], titanium carbide (Ti_2_C) [91], and Fe^3+^ [211].

Strategies to ensure survival, differentiation, cell attachment, and engraftment have been achieved through cell preconditioning. Preconditioning involves cell culture under hypoxic conditions [82], serum starvation [83], with cytoprotective molecules like antioxidants that reduce the oxidative stress and enhance their regenerative potential [81], under vasculogenic conditioning where cell types shift their phenotype to proregenerative cells [79], and incubation with a combination of human connective tissue growth factor (C‐terminal, fourth domain peptide; CTGF‐D4) and insulin significantly to increase the engraftment [80], as well as electrophysical (mechanical, electrical, or electromechanical) stimulation [75–78, 83], which mimics the dynamics occurring in the infarcted myocardium. This strategy has been shown to increase proliferation and differentiation of MSCs and ADSCs into cardiac cells, along with upregulation of survival and angiogenesis signaling pathways.

Recently, a variety of nanomaterials have been developed for the treatment of AMI. Sustained drug release systems prolong the retention of therapeutic drugs within target tissues to alleviate the need for repeated drug administration. Nanodrug technology has allowed for delayed quantitative drug release and reduced the amount of medication required, improving the treatment strategy. This review identified various controlled drug release strategies to address the oxidative environment [92–95] and prevent fibrosis [96–98] and remodeling of the ventricle [99–101] and delivery of factors that allow differentiation of the cell source [102], as well as general proposals for drug delivery system designs [212–214].

#### 3.1.6. Stage 6: Cardiac Regeneration

A variable time frame of interaction between the therapy and the injury area has been reported; 63% (*n* = 213) of the articles analyzed the effects of therapy at 4 weeks, 19% (*n* = 64) after 5 weeks, and 18% (*n* = 62) within 4 weeks after the administration of therapy.

Making a comparison of the effectiveness of TD between papers is complex, due to the variability of the TD design. Regulation of the inflammatory environment during the early remodeling phase, using constructs or cell‐free scaffolds, reduces fibrosis [44] and increases cell survival [155] and angiogenesis in the injury area [215]. The reduction of ECM elements that affect adhesion has been addressed with novel strategies in TD design, which seek to mimic the ECM microenvironment or ensure anchoring of the TD to the host tissue, which has resulted in improved cell communication and adhesion [184, 216, 217]. Other strategies that have aimed at reducing oxidative stress [158], providing elements of the ECM [139, 156, 161], and promoting fibroblast recruitment [28] are reported to have limited the extent of injury damage and facilitated the transition to the formation of granulation tissue. TD vascularization strategies are used to enhance cell retention and survival [53]. The use of prevascularized devices, self‐assembly peptides, and cell aggregates that secrete angiogenic factors has been more effective than the implantation of only cells that differentiate into blood vessels. Cell retention values of 50% to > 80% compared with monolayer grafting or cell‐only administration [147, 148, 183] and a decrease of ≤ 50% in cell death [218] have been reported. Moreover, the induction of new blood vessels in the periphery of the injured myocardial area improved cell retention and survival [55, 150]; thus, the identification of blood vessels in the center of the injury is of special interest. TDs designed with conductive materials promoted synchronous contraction of cardiac cells [9, 85, 219]. Their use increased the expression of connexin 43, thereby facilitating conduction of the electrical impulse in the infarcted area [9, 10, 85]. The electrical coupling of the TD with the native myocardium and the activation of CM contraction at the appropriate time improved LVEF [219], and this effect has been reported to last for long periods of time [91].

Despite the difficulty of comparing the efficacy reported during the preclinical validation of the different TDs, advances in reducing the size of the lesion and in the recovery of ventricular functionality can be explored.

**Figure 5:**
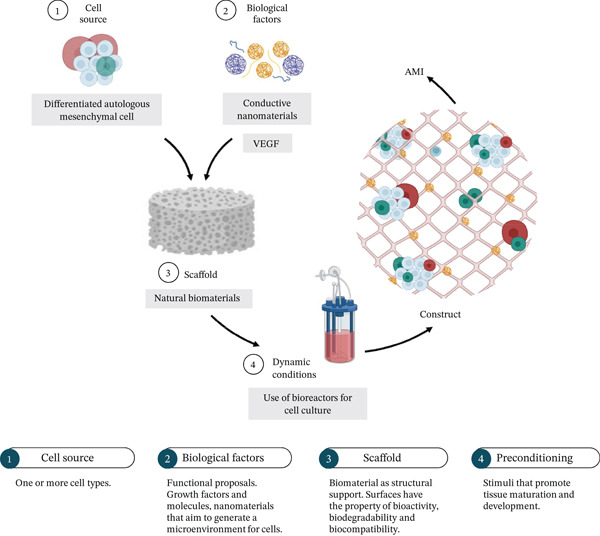
Preclinical efficacy of TD in cardiac regenerative therapy. Model that summarizes the techniques used so far to obtain the best results during preclinical validation of cardiac TD. The proposals have been functionalized in order to reduce cell loss by favoring angiogenesis and decreasing the inflammatory environment.

An effective approach to tissue engineering involves the development of scaffolds with cells (construct). 3D structures induce angiogenesis by affecting cell migration, proliferation, and differentiation, enabling the implanted tissue to survive effectively, thereby directionally repairing damaged tissue. Tissue scaffolds are interconnected porous structures that stimulate cell metabolism by simulating extracellular environments and provide appropriate mechanical support to maintain structural stability in vivo; at the same time, tissue scaffolds should be biodegradable and ideally compatible with cell and tissue regeneration. Moreover, the use of biomaterials enables the incorporation of growth factors or molecules that promote a suitable environment for cell delivery, differentiation, and survival (Figure 5).

According to our analysis, 16% of the constructs that report a decrease of up to 70% in the size of the infarct include those aimed at promoting electrical conductivity [16, 85, 220], between the myocardium and the tissue, and those that serve the ROS produced after AMI [173, 221]. Twenty‐nine percent of the studies managed to reduce the size of the infarct between 50% and 60% compared to their AMI group. Finally, 55% of the constructs report a decrease of less than 40% in infarct size, where the functionalized constructs represent the strategies with the best results [58, 85, 140].

The LVEF is a variable that is difficult to modify; only 9% of the constructs report an increase greater than 60% compared to the AMI [28, 222], and 22% of the papers report an increase in LVEF of 50% compared to the AMI group [13, 46, 58], while 69% of the papers report an increase of less than 40% compared to the infarcted group [51, 85, 140, 185].

It is important to mention that based on the reports analyzed in our database, they report the increase in one variable with respect to the other. They report a considerable decrease in infarct size, but a poor increase in LVEF and vice versa [28, 58, 85]. Therefore, important challenges are observed in the development of therapies in cardiac tissue engineering.

According to our observations, the following must be taken into account for the design of the constructs (Figure 5). The selection of biomaterials must consider the stringent requirements placed on cardiac tissue [186], which are necessary not only to support cell attachment and alignment but also to transmit charge, provide physiologically relevant stiffness, and be replaced over time by ECM proteins secreted by cells. The TD should allow CMs to develop a mature contractile phenotype and to communicate with adjacent cells.

In native cardiac tissue, the ECM provides this crucial physiological environment [62]. In addition, it is known that resident macrophages participate in orchestrating the heart rate [45]. In this sense, the mechanical properties, porosity, topography, electrical properties, degradation, bioactivity, and biocompatibility of TD are related to cell migration, including various cell types, cell adhesion, and changes in cell morphology [18, 174, 179]. These responses affect cell growth, differentiation, and proliferation and facilitate nutrient supplementation.

The “ideal” biomaterial will be one that has a biochemical composition, structure, and function like that of the native cardiac ECM. Any tissue engineering technology must recapitulate the target tissue in vitro to maximize the efficacy of tissue engineering approaches. Overall, it has been shown that the greatest efficacy is achieved with functionalized TDs [3, 25, 27, 187] (Figure 5), especially when implanted early in granulation tissue formation [19, 174]. Although recent data are promising, further research is needed to verify the effects of using functionalized strategies in the various stages of cardiac remodeling.

Figure 4 shows the process flowchart for preclinical validation of cardiac regenerative therapy. The use of large animal models for the evaluation of lesion formation in later stages should be useful to compare with clinical settings. Once sufficient evidence of safety and efficacy has been established in animal models, clinical trials will be able to evaluate the long‐term (years) effect of the therapy by focusing on measurements of cardiac function.

#### 3.1.7. Stage 7: Frequently Reported Variables

Therapeutic efficacy has been evaluated through the analysis of cardiac function, infarction size, increase in viable myocardium, promotion of neovascularization, and suppression of inflammatory response. Table 6 shows a methodological comparison of LVEF and myocardial lesion quantification based on the most reported techniques for their analysis in preclinical and clinical studies.

**Table 6:** Methodological comparison of left ventricular ejection fraction assessment and myocardial lesion quantification.

Aspect	LVEF analysis	Myocardial damage analysis
Main objective	Assess global systolic function of the left ventricle.	Quantify structural damage, loss of viable tissue, and lesion size.
What it measures	Left ventricular ejection fraction (LVEF), cardiac volumes, and dimensions.	Percentage of fibrosis, ventricular wall thickness, and infarct/lesion size.
Physiological relevance	Indicates how efficiently the heart pumps blood.	Directly associated with the amount of necrotic tissue and remaining functional myocardium.
Most common methods of analysis	Echocardiography (most common in murine models); cardiac MRI (gold standard, less used in small animal studies); cardiac catheterization (rare).	Routine histology; fibrosis staining; wall thickness measurement; direct lesion area quantification.
Advantages of each method	Echocardiography: noninvasive, reliable, widely available; suitable for longitudinal monitoring.CMRI: high precision.	Routine histology: directly measures damage; fibrosis staining provides detailed information on nonviable tissue; lesion size correlates with functional loss.
Limitations	Echocardiography has lower precision than CMRI; CMRI is expensive; catheterization is invasive and complex.	Variability due to lack of protocol standardization; many studies report only one parameter.
Standardization recommendation	Systematic use of echocardiography, and report LVEF as a standard parameter.	Report lesion size as the primary variable; complement with fibrosis and wall thickness.
Clinical vs. preclinical studies use	Clinical: echocardiography and CMRI are widely used; preclinical: conventional ultrasound predominates.	Clinical: fibrosis strongly predicts outcomes; preclinical: histology predominates, standardization needed.

Ventricular function is assessed by quantifying the rate of change of LVEF, fractional shortening, and ventricular internal diameter. Most of the studies included representative echocardiograms showing improved ventricular function [31, 67, 76, 123]. Important measurements such as the percentage of damaged area, injury thickness, and percentage of collagen deposition are usually reported [151, 223].

The most frequently reported molecular biomarkers of cardiac remodeling include neovascularization, differentiation, adhesion, death, macrophage activation, damage, and others. Smooth muscle alpha‐actin and alpha actinin [44, 91, 156], von Willebrand factor (vWF), isolectin, and CD31 are used to assess the promotion of neovascularization [28, 147], and interleukins (IL‐1*β* and 6), TNF‐*α*, and CD68 are associated with the inflammatory process [5, 155, 184].

In addition, preclinical validation studies often report other features of the therapy, such as TD characterization, biocompatibility, biodegradation, inflammatory response, newly formed blood vessels, and cell adhesion. Cell adhesion is important for those therapies that are aimed at improving delivery routes and construct design [10, 23, 28, 155, 184].

#### 3.1.8. Limitations of Published Studies

Some studies did not provide information about strain (12%, *n* = 42), weight (38%, *n* = 128), gender (32%, *n* = 85), or sample size (16%, *n* = 54), which may hinder its reproducibility. A large variability in sample size per study group was observed among the different publications. These differences should be considered when analyzing the results and conclusions.

Less than 5% (*n* = 14) of papers included a biocompatibility test prior to AMI induction, or they document it in a different paper. However, the biocompatibility test provides an opportunity for continuous improvement in the development of a TD.

Standardization of the infarction model requires an exhaustive process. The time and site of occlusion lead to variations in the injury size [224] and in the final LVEF values [78, 131]; therefore, it is essential to describe the characteristics associated with the development of the infarction model as part of the methodology. Furthermore, < 30% of the literature reviewed set the inclusion criteria based on morpho‐pathological and physiological changes in the infarcted groups (LVEF, injury size, or fractional shortening after induction). A value of 30%–40% of LVEF or an injury size of ~40% of the LV area is suggested as the inclusion criterion for the infarcted group. These values increase the probability of survival of the animals throughout the study [44] and represent the cutoff values for the diagnosis of heart failure in humans [225]. A standardized starting value allows homogeneous comparison of the feasibility of cardiac TDs between studies.

TDs are not always able to restore normal cardiac function; 29% of the publications reported final LVEF values associated with ventricular dysfunction (< 40% LVEF), 25% at risk of dysfunction (40%–50% LVEF), and only 46% presented final values within the normal range (> 55% LVEF). This pattern could be modified depending on the time and cell retention capacity of the proposed therapy.

Another limitation to consider when comparing the effectiveness of therapy is to consider the clinical scenario. Clinical trials to validate stem cell grafts reported administration of the therapy within 24 h to 27 days after percutaneous coronary intervention [11, 226, 227]. This period corresponds to the inflammatory and proliferative phases of human ventricular remodeling [130]. The timing of therapy administration should consider the stage of ventricular remodeling in relation to the clinical time of intervention of the infarcted patient. Studies that evaluate the effect of scaffold implantation at different times (early and late phase) have documented that those values associated with infarct size, LVEF, and FS improve when the scaffold is implanted in the late phase of remodeling [130, 228]. However, values associated with the implantation of a cellular source are not reported; variables such as cell death and cell proliferation, among others, may be affected when the therapy is implemented in a late phase.

Among the essential variables to report, it is necessary to consider the change in infarct size, the quantification of blood vessels, and, if possible, the incorporation of cardiac function.

## 4. Conclusions and Future Perspective

This review identified the relevant aspects of each stage of preclinical validation, with the aim of promoting systematization and experimental reproducibility to obtain robust scientific evidence. Special emphasis should be placed on statistical representativeness criteria, inclusion of the clinical characteristics, type of AMI induction, and time of progression of the cardiac injury. Based on the laboratory’s prior knowledge of its TD, it would be appropriate to produce evidence showing the safety of the TD and choose the delivery route and the time of cardiac regeneration after implantation. The choice of variables should preferably include the evaluation of anatomical and functional parameters, such as infarct size and LVEF. This will enable systematic comparisons to be made on the feasibility of each proposed TD.

It is understandable for the management of the models in the laboratory that the TDs are tested at the moment of inducing the AMI (early stages of cardiac remodeling). However, the myocardial microenvironment is highly complex following AMI, and the success of the therapy is related to its ability to elicit a specific biological response in adverse conditions similar to those of the late stage of remodeling. Most research efforts have focused on securing the TD to the host tissue, which can be achieved by using natural materials and functional proposals. The electrical communication of the artificial myocardium and the anti‐inflammatory effects of TDs have not been thoroughly explored. The preclinical evidence indicates that it is currently designated to address only a few aspects of this complex system. The current design of TDs must include not only the search for cell survival and retention but also mechanical properties to mimic those of natural myocardial tissue or by designing anchoring structures, promoting an adequate microenvironment for cells, reducing the inflammatory process, and forming blood vessels, with the materials used already approved for medical use.

Therefore, more functional composite proposals will be developed in the future. The correct systematization of the preclinical validation of these proposals will allow their transfer in large animal models and in the clinical setting.

According to the literature reviewed, we propose functionalized constructs using bioreactors for cardiac therapy (Figure 5). TDs should employ differentiated autologous mesenchymal stem cells, seeded on a conductive scaffold made from natural biomaterials. Functionalization of the scaffold can focus on blood vessel formation through the controlled release of VEGF. Cell conditioning using dynamic culture (bioreactors) will allow the generation of tissue with a mature cardiac phenotype.

## Funding

The study was funded by Protocols HIM/2020/059 and HIM/2022/040 from Federico Gomez Children’s Hospital of Mexico and CONACYT.

## Disclosure

This work was conducted as part of the doctoral studies of student Nancy G. Viveros‐Moreno, with funding from CONACYT.

## Conflicts of Interest

The authors declare no conflicts of interest.

## Data Availability

Data sharing is not applicable to this article as no datasets were generated or analyzed during the current study.
